# Intelligent Reflecting Surface Assisted Multi-User Robust Secret Key Generation for Low-Entropy Environments

**DOI:** 10.3390/e23101342

**Published:** 2021-10-14

**Authors:** Yuwei Gao, Dengke Guo, Jun Xiong, Dongtang Ma

**Affiliations:** College of Electronic Science and Technology, National University of Defense Technology, Changsha 410072, China; gaoyuwei14@nudt.edu.cn (Y.G.); guodengke18@nudt.edu.cn (D.G.)

**Keywords:** key generation, physical layer security, multi-user

## Abstract

Channel secret key generation (CSKG), assisted by the new material intelligent reflecting surface (IRS), has become a new research hotspot recently. In this paper, the key extraction method in the IRS-aided low-entropy communication scenario with adjacent multi-users is investigated. Aiming at the problem of low key generation efficiency due to the high similarity of channels between users, we propose a joint user allocation and IRS reflection parameter adjustment scheme, while the reliability of information exchange during the key generation process is also considered. Specifically, the relevant key capability expressions of the IRS-aided communication system is analyzed. Then, we study how to adjust the IRS reflection matrix and allocate the corresponding users to minimize the similarity of different channels and ensure the robustness of key generation. The simulation results show that the proposed scheme can bring higher gains to the performance of key generation.

## 1. Introduction

CSKG is different from traditional key-based upper-layer encryption [1], which is a physical layer security(PLS) scheme based on information theory. Due to the time-varying, short-term reciprocity, and space–time uniqueness of the shared wireless channel, legitimate nodes can use the channel information as a natural source to extract secret keys without key exchange [2]. However, CSKG schemes are limited by randomness in the channel conditions, which depends on the dynamics in the wireless environment. Therefore, it is impractical to obtain sufficiently random keys under a low-entropy environment. This problem has always been a challenge [3], which especially affects a wide range of wireless applications with limited mobility, that is, the channel tends to be static. This issue has been studied in the literature and various solutions have been proposed [4,5,6], including utilizing multiple-input multiple-output (MIMO) antennas [4], beamforming [5], and deploying friendly jamming [6], but the above methods greatly increase energy and hardware deployment costs, which means that they can not be applied on the existing CSKG system widely.

Intelligent reflecting surface is an artificial plane with digitally controllable electromagnetic reflection behavior, which is composed of a large number of individually adjustable reflective elements [7]. Due to its passive reflective characteristics, IRS can work without active radio frequency (RF), and thus it can work in full-duplex systems with low energy consumption. In addition, IRS has a great innovation potential to control the propagation of radio under the condition of relatively low hardware complexity. In recent years, IRS has also been considered to improve the physical layer security of wireless communication networks [8,9], pointing to the promising future of the wireless communication material. However, most of these works focus on the research of secure transmission, i.e., using passive beamforming to maximize the signal-to-noise ratio (SNR) difference between the legal and eavesdropping channel, and few works consider the use of IRS for wireless key generation.

Since IRS can configure the wireless channel through passive reflection, it has great potential in improving key extraction performance [10]. In terms of applying IRS to optimize key extraction over a low-entropy channel, some papers have recently proposed corresponding schemes [11]. A low-cost IRS-assisted channel key extraction prototype system was also recently designed in [12], which proved that using IRS for key extraction is a feasible solution.

However, the existing literature only considers the point-to-point key extraction over a static channel. When the scenario is extended to a multi-user application, and the users are relatively close, the key generated by each user is very relevant. This will damage the security and the efficiency of the key generation. This paper considers channel key extraction in multi-user downlink scenarios.

The usual method is assigning an IRS random coefficient to each user separately, so that the multi-user, static channel-related problems can be solved. However, in the actual key extraction process, each user needs to negotiate with the base station, and a high signal-to-noise ratio can facilitate the negotiation interaction. Therefore, each IRS random coefficient set needs to be effectively matched with the user channel. Based on the above considerations, this paper studies the key generation scheme that combines user selection and IRS random change in a multi-user downlink scenario. The simulation results show that, compared with the no-IRS and no-user-selection scheme, it can bring performance improvement in the robustness of information interaction and the key disagreement rate (KDR).

The main structure of this paper is as follows. Section 2 gives the IRS-assisted multi-user communication system model. Section 3 proposes the joint user allocation and IRS reflection parameter adjustment scheme. Section 4 gives the simulation results and Section 5 gives the conclusion of this paper.

Notations: Throughout our discussions, the distribution of complex Gaussian random variables with mean 0 and variance $\sigma^{2}$ are denoted by $\sim CN(0,\sigma^{2})$. ${\mathbb{C}}^{M\times N}$ denotes the space of *M* × *N* complex-valued matrices.

## 2. System Model

**Figure 1:**
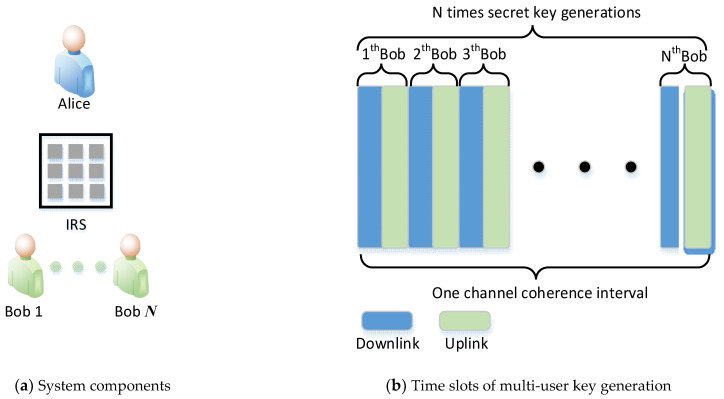
System model of key generation.

This article considers a situation in which the channel information of illegal eavesdropper is unknown. The components of the system are described in Figure 1a, which included a single Alice, a single IRS, and *N* Bobs. The IRS was equipped with L passive reflective elements, and the Alice and all Bobs were equipped with a single antenna. This paper assumed that Alice and IRS fully knew the global channel state information (CSI) on all the channels involved. Without the loss of generality, we considered a common communication scenario in which all nodes were stationary and Bobs were adjacent, which means that the channels are highly similar. Legitimate nodes generally obtain secret keys through channel probing [13,14], quantitation [15,16], information negotiation [17,18], and privacy amplification [19,20]. As shown in Figure 1b, Alice and *N* Bobs performed the key extraction in turn within one channel coherence interval, and the time interval for each round of the key extraction was fixed.

Secret key capacity means the maximum achievable rate of the key generation. As shown in Figure 1, we assumed that user distribution was uniform; then we took Bob 1 as an example to analyze the key capacity without IRS. $h_{B_{1}}$ and $h_{B_{1}^{\prime }}$ represent the uplink and downlink channel information obtained by Alice and Bob1, respectively. $h_{B_{2}},h_{B_{3}},\cdots ,h_{B_{N}}$ represent the channels of other Bobs. Therefore, we can express the key capacity of Bob1 as the mutual information between $h_{B_{1}}$$h_{B_{1}^{\prime }}$ and minus the leak to other channels:

(1) $$I_{B^{1}}=I\left(h_{B_{1}};h_{B_{1}^{\prime }}|h_{B_{2}},h_{B_{3}},\cdot \cdot \cdot ,h_{B_{N}}\right)$$

And we can consider the information leaked to other channels as a whole unit, namely, we let $h_{E}=h_{B_{2}},h_{B_{3}},\cdot \cdot \cdot ,h_{B_{N}}$. Equation (1) can be derived as follows:

(2) $$\begin{array}{cc} I_{B^{1}} & =I\left(h_{B_{1}};h_{B_{1}^{\prime }}|h_{E}\right)\\ & =H\left(h_{B_{1}}|h_{E}\right)+H\left(h_{B_{1}^{\prime }}|h_{E}\right)-H\left(h_{B_{1}};h_{B_{1}^{\prime }}|h_{E}\right)\\ & =H\left(h_{B_{1}},h_{E}\right)+H\left(h_{B_{1}^{\prime }},h_{E}\right)-H\left(h_{E}\right)-H\left(h_{B_{1}},h_{B_{1}^{\prime }},h_{E}\right)\\ & \overset{a}{=}H\left(h_{B_{1}},h_{E}\right)-H\left(h_{E}\right) \end{array}$$

where H(·) represents the corresponding entropy. Since (a) holds because of channel reciprocity, $h_{B_{1}}$ and $h_{B_{1}^{\prime }}$ are highly similar. It can be seen from Equation (2) that, as the channel similarity of each user increases, the difference between the two items in the last row becomes smaller. This means that the high similarity between users’ channels will greatly reduce the key capacity. Furthermore, it shows that, when a user’s channel is more independent of other channels, the achievable key rate is faster, which means that we can increase the key generation rate by reducing the channel correlation between multiple users.

**Figure 2:**
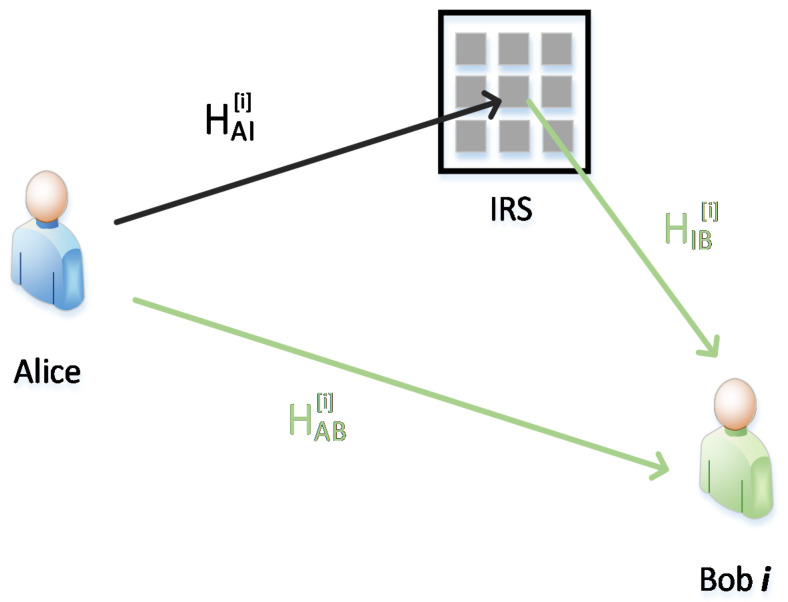
IRS-aided Communication System.

The channel model of a single user is showed in Figure 2. Alice and Bob are network nodes that aim to extract the secret key from the shared channel. To ensure the reciprocity between the downlink and uplink channels within coherent time, all communication nodes adopted a half-duplex working mode and time-division-duplex (TDD) communication style. Furthermore, this paper assumed channel coefficients of Alice–Bobs links ($h_{AB^{i}}$), Alice–IRS link ($h_{AI}$), and IRS–Bobs links ($h_{IB^{i}}$) are satisfied as $h_{AB^{i}}\sim CN(0,\sigma_{AB^{i}}^{2})$, $h_{\Delta }\sim CN(0,\sigma_{h_{\Delta }}^{2})$$\Delta \sim (AI,IB^{i})$, where i means i-th Bob, i=1,…N.

The signal received can be expressed as:

(3) $$y_{B}^{i}=(h_{IB^{i}}^{H}\Theta h_{AI}+h_{AB^{i}}^{})x+z_{B^{i}}$$

For the IRS channel, similarly to [21], we assumed that $\Theta =\mathrm{diag}\left\{\left[\beta_{1}e^{j\phi_{1}},\cdot \cdot \cdot ,\beta_{m}e^{j\phi_{m}},\cdot \cdot \cdot ,\beta_{L}e^{j\phi_{L}}\right]\right\}$ denotes the diagonal amplitude-phase shifting reflecting coefficient matrix of IRS, where $\beta_{m}$ and $\phi_{m}$ are the amplitude and phase shifts, respectively, on the incident signal by *m-th* element, m=1,…L. L is the IRS reflector number.$z_{B^{i}}\sim CN(0,\sigma_{B^{i}}^{2})$ denotes the noise at Bob i.

## 3. Joint User Allocation and IRS Reflection Parameter Adjustment Scheme

To solve the key similarity problem, we firstly disturbed the channel using *N*-uncorrelated IRS reflection matrices to *N* Bobs and then obtained keys with lower similarity. Note that, in the following, we used $\Theta^{j}$ to represent the *j*-th IRS reflection matrix j=1,⋅⋅⋅,N.

This paper considers two continuous and discrete classic modes of IRS reflection matrix generation. Under continuous conditions, we assumed that the IRS reflection matrix obeyed the complex Gaussian distribution, which maximizes the entropy over all distributions with the covariance constraint [22]. In the discrete case, we assumed that the reflection matrix generated satisfied the full power reflection condition, namely, $\beta_{m}=1$. The reflection element of the IRS only adjusted the phase to −π or π, namely, $\Theta =\mathrm{diag}\left\{\left[e^{j\phi_{1}},\cdot \cdot \cdot ,e^{j\phi_{m}},\cdot \cdot \cdot ,e^{j\phi_{L}}\right]\right\}$ $\phi_{m}\in \{-\pi ,\pi \}$.

In addition, Alice and Bob had multiple information exchanges in the key generation. The reliability of the obtained information was more precise when the channel state was better. For example, in the channel detection stage, under the same channel detection frame design, the larger channel signal-to-noise ratio (SNR), the more accurate the channel estimation that was obtained, which ensured the robustness of subsequent key extractions. Therefore, we considered user allocation based on the generated IRS reflection matrix set to enhance the reliability of key generation. Channel capacity means the maximum achievable information rate. h represents the CSI obtained by receiver. Therefore, the channel capacity can be expressed as follows:

(4) $$R=\log_{2}(1+\frac{{\left\|h\right\|}^{2}}{\sigma_{h}{}^{2}})$$

where $\sigma_{h}^{2}$ denotes variance of the channel noise. We can take Equation (3) into Equation (4) to obtain the channel capacity of the system:

(5) $$R_{B}^{i,j}{=\log}_{2}(1+\frac{{\left\|h_{IB^{i}}^{H}\Theta^{j}h_{AI}+h_{AB^{i}}^{}\right\|}^{2}}{\sigma_{B^{i}}^{2}})$$

Equation (4) shows that the channel capacity of the IRS-aided system can be adjusted by the reflector $\Theta^{j}$. Then, we can adjust the IRS reflector to improve information exchange reliability. The overall optimization problem can be expressed as follows:

(6) $$\max\underset{i,j}{\sum }R_{B}^{i,j}\;s.t\Theta^{j}\in \Theta \;\;i,j=1,\ldots ,N$$

To find out the optimal parameter configuration of IRS, we propose the corresponding configuration algorithm. Firstly, the generated *N* IRS reflection matrices are paired with *N* receivers to obtain the channel capacity under the corresponding pairings. Secondly, we obtain a N×N value matrix to be sorted, where the *i*-th row is the result of the *i*-th Bob that sequentially matches all IRS reflection matrices. Then, the first pair is the pair with the largest value. In order to sufficiently reduce the randomness between keys, a reflection matrix is allocated to only one receiver. Finally, in the remaining pairs without elements of the last pair, we find the next pair with the largest value, and so on, until all reflection matrices are allocated. As a result, the allocation process of the proposed scheme can be summarized as shown in Algorithm 1.

**Algorithm 1** Rank algorithm of IRS allocation for Bobs scheduling.**Input:**$R_{B}^{\left(i,j\right)}$ **Output:***pair* pair=∅ **for** loop = 1:N $\left(i,j)=\underset{i,j?[1,N]}{\mathrm{argmax}}(R_{B}^{\left(i,j\right)}\right)$ $R_{B}^{}=R_{B}^{}\backslash (R_{B}^{\left(i,:\right)}UR_{B}^{\left(:,j\right)})$ pair=pairU(i,j) % Pair relationship between IRS reflection matrix and Bob **end**

## 4. Simulation and Numerical Results

We analyzed the proposed scheme through Monte Carlo simulation. The number of Bobs is set to 16, and all channels in the model—$h_{AB^{i}}$, $h_{AI}$, and $h_{IB^{i}}$—are set as complex Gaussian channels conforming to CN(0,1). To make the results more accurate, we performed 10,000 simulations for each parameter point.

**Figure 3:**
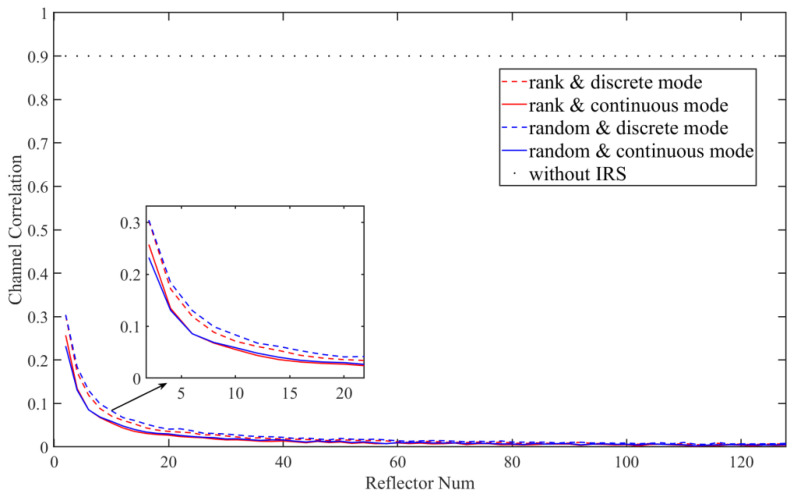
Comparison of the IRS-random algorithm and IRS-rank algorithm on channel correlation. The ‘rank & discrete mode’ gives the performance line using our rank algorithm under the discrete IRS mode; ‘random & continuous mode’ gives the performance line using random algorithm under the ‘continuous IRS mode’.

We adopted the Pearson correlation coefficient [1] to analyze the impact of the IRS on the correlation of adjacent multi-user channels. Without IRS, we set the coefficient between user channels as 0.9. Figure 3 shows the correlation coefficients of channel measurement values between multiple Bobs under different reflector numbers *L*(1~128). It can be seen that, compared with the system without IRS, the correlation between Bobs of the IRS-assisted system is greatly reduced, which effectively improves the overall key extraction efficiency and key security. In addition, we also compared our scheme with the random sorting algorithm, which mainly assigns the generated matrixes to each user randomly. Thus, we can see that under the two IRS modes, when the reflector number reaches about 20, the disturbance of the channel tends to be smooth and the discrete mode has a relatively low degree of disturbance to the channel.

**Figure 4:**
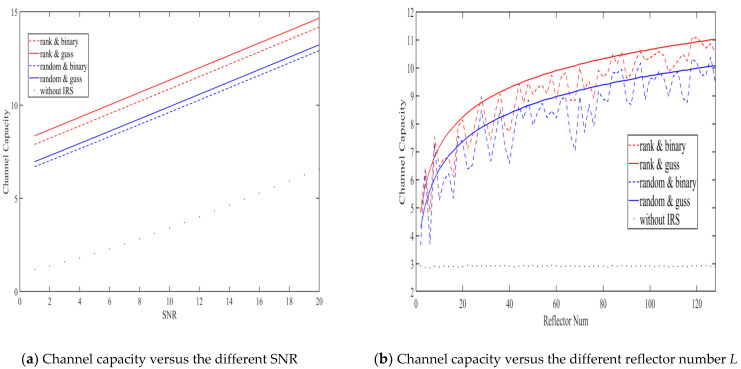
Comparison of the IRS-random algorithm and IRS-rank algorithm on channel capacity.

In addition, we set the number of reflectors as 128, and compared the effect of the IRS on the improvement of the channel capacity under the condition that the user channel SNR is 1 to 20 in Figure 4a. Next, we fixed the SNR to be 10, and analyzed the effect of the IRS reflector number L(1~128) on channel capacity in Figure 4b. We noted that the channel capacity in the figure is normalized, that is, the average channel capacity of each user channel.

As shown in Figure 4a, compared with the system without IRS, the channel capacity of the IRS-assisted system was greatly improved, which means that the proposed scheme guarantees the improvement of information exchange reliability in the key generation process. It can also be observed that our proposed scheme performs better than random algorithms, i.e., the ‘rank & discrete mode’ and ‘rank & continuous mode’, and that the ‘continuous IRS mode’ has better performance.

As shown in Figure 4a, we can see that, as the scale of the IRS increases, the channel capacity continues to rise, and the uptrend tends to be smooth. It can also be observed that the ‘continuous IRS mode’ is essentially the upper limit of the performance of the discrete mode.

We also compared the key disagreement rate performance of the different schemes that adopt multi-bit adaptive quantization with equal probability [15]. We set the reflector number as 128, and analyzed the influence of IRS on KDR when the user channel signal-to-noise ratio is 1 to 10. The key disagreement rate is assumed as $\;K_{dis}/K_{total}$, where $K_{dis}$ is the inconsistent key bits number and $K_{total}$ is the total key bits number obtained by Alice and Bobs in each channel detection.

**Figure 5:**
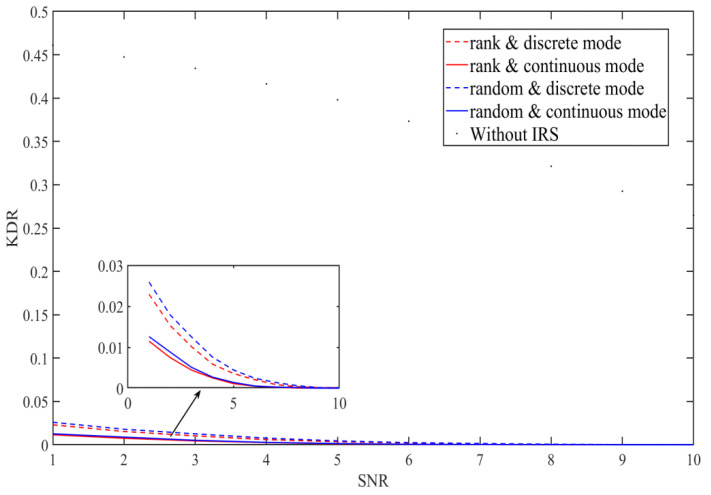
Comparison of the IRS-random algorithm and IRS-rank algorithm on the key disagreement rate.

Since our solution improves the channel environment during the key generation process, there is less noise interference on communication parties and the inconsistency of the keys generated by the two parties can be reduced. As shown in Figure 5, compared with the system without IRS, the KDR of the IRS-assisted system is greatly reduced. It can be seen that the KDR of the IRS-rank algorithm is better than the IRS-random algorithm. This means our scheme can obtain the initial channel keys with a lower key disagreement rate, which makes information coordination easier to implement in the process of key generation.

## 5. Conclusions

The high similarity of the generated secret key between adjacent multi-users in a low-entropy channel results in low key generation efficiency. This paper proposed a new IRS-assisted key generation scheme, which mainly studied the problem of user allocation under the random variation of IRS parameters. Our scheme can greatly reduce the similarity of the adjacent multi-users’ channel to improve the efficiency of key generation. Morover, it can ensure the reliability of information exchange in the key generation process, which makes our scheme able to reduce the key inconsistency. The simulation results showed that our proposed scheme greatly improves the performance of the key extraction process.

## Data Availability

Not applicable.
